# Optical Methods for Brain Tumor Detection: A Systematic Review

**DOI:** 10.3390/jcm13092676

**Published:** 2024-05-02

**Authors:** Gustav Burström, Misha Amini, Victor Gabriel El-Hajj, Arooj Arfan, Maria Gharios, Ali Buwaider, Merle S. Losch, Francesca Manni, Erik Edström, Adrian Elmi-Terander

**Affiliations:** 1 Department of Clinical Neuroscience, Karolinska Institute, 171 77 Stockholm, Sweden; gburstrom@gmail.com (G.B.); misha.amini@stud.ki.se (M.A.); arooj.arfan@stud.ki.se (A.A.); mcg02@mail.aub.edu (M.G.); ali.buwaider@stud.ki.se (A.B.); erik.edstrom.1@ki.se (E.E.); 2 Department of Biomechanical Engineering, Faculty of Mechanical Engineering, Delft University of Technology, 2627 Delft, The Netherlands; 3 Department of Electrical Engineering, Eindhoven University of Technology (TU/e), 5612 Eindhoven, The Netherlands; f.manni@tue.nl; 4 Capio Spine Center Stockholm, Löwenströmska Hospital, 194 80 Upplands-Väsby, Sweden; 5 Department of Medical Sciences, Örebro University, 701 85 Örebro, Sweden; 6 Department of Surgical Sciences, Uppsala University, 751 35 Uppsala, Sweden

**Keywords:** neuro-oncology, optical coherence tomography, diffuse reflectance spectroscopy, hyperspectral imaging, Raman spectroscopy, accuracy, review

## Abstract

**Background:** In brain tumor surgery, maximal tumor resection is typically desired. This is complicated by infiltrative tumor cells which cannot be visually distinguished from healthy brain tissue. Optical methods are an emerging field that can potentially revolutionize brain tumor surgery through intraoperative differentiation between healthy and tumor tissues. **Methods:** This study aimed to systematically explore and summarize the existing literature on the use of Raman Spectroscopy (RS), Hyperspectral Imaging (HSI), Optical Coherence Tomography (OCT), and Diffuse Reflectance Spectroscopy (DRS) for brain tumor detection. MEDLINE, Embase, and Web of Science were searched for studies evaluating the accuracy of these systems for brain tumor detection. Outcome measures included accuracy, sensitivity, and specificity. **Results:** In total, 44 studies were included, covering a range of tumor types and technologies. Accuracy metrics in the studies ranged between 54 and 100% for RS, 69 and 99% for HSI, 82 and 99% for OCT, and 42 and 100% for DRS. **Conclusions:** This review provides insightful evidence on the use of optical methods in distinguishing tumor from healthy brain tissue.

## 1. Introduction

In brain tumor surgery, the extent of resection of tumors has significant implications on relapse, overall survival, quality of life, and the need for adjuvant or salvage therapy [1,2]. The burden of locoregional tumor recurrence resulting from tumor remnants after surgery is vast and often inevitable in malignant tumor types [3]. Consequently, gross total resection is generally the aim of brain tumor surgery, with exemptions in eloquent areas of the brain. However, tumor borders are rarely sharp, as infiltration into healthy brain tissue is common in malignant brain tumors. The inability to accurately visualize tumor borders during operation hence threatens the balance between gross total resection of tumor tissue and preservation of healthy brain tissue [4,5]. Technologies that aim to optimize the extent of resection by offering tissue demarcation capabilities are hence highly relevant to the field. Image-guided neuronavigation, 5-aminolevulnic acid (5-ALA) fluorescence-guided surgery, intraoperative ultrasound guidance, and intraoperative MRI are some of the most employed technologies for this purpose [5,6]. Whilst all these technologies have various degrees of reported benefit in optimizing the extent of resection during surgery [7,8,9], they offer macroscopic visibility only. 

Neuronavigational techniques rely on preoperative MR images, leading to decreased accuracy as brain shift and brain deformation during surgery cause tumor boundaries to shift [10]. Intraoperative MRI solves the brain shift problem but faces challenges due to poor spatial resolution, high cost and increased operation time [11]. Intraoperative ultrasound is affordable but is limited due to image artefacts and challenging patient positioning, making it difficult to standardize and interpret [12]. Finally, fluorescence-guided techniques such as 5-ALA are invasive, show variable uptake of contrast among patients [13] and have low utility in certain anatomical locations [14]. These fluorescence-guided techniques are most accurate when targeting contrast-enhancing disease with high cellularity, making them suboptimal in many patient groups where resection of non-enhancing tissue has been shown to improve long-term survival [15]. While improvement of these technologies may be an option, the development of more advanced, and reliable tools certainly merits further investigation. 

Intrinsic optical imaging techniques are emerging as one of the most promising frontiers in the field [16,17,18,19]. These minimally invasive, label-free techniques directly detect tumor infiltration based on the biochemical properties of the tissue and, in some cases, with sub-micron resolution. Each tissue has an individual optical “fingerprint” depending on its microstructure, cellular and mitochondrial density, molecular composition, presence of natural pigments (e.g., hemoglobin, beta-carotene, melanin), and pathological or physiological factors such as vascularization or necrosis, etc. [20]. There are various tools used for the detection of different parameters of light reflection, all of which utilize different intrinsic optical concepts.

These methods have long been applied in preclinical settings [21]. The more recent advances in computational power, machine learning, data processing and optical fiber technologies have now propelled the field towards human-based clinical studies [22,23]. Some of the most widely used and currently investigated optical methods specifically for applications in brain tumor surgery include Multispectral/Hyperspectral Imaging, Raman Spectroscopy, Optical Coherence Tomography and Diffuse Reflectance Spectroscopy, each individually presented below. 

### 1.1. Raman Spectroscopy

Of the intrinsic optical methods, Raman spectroscopy is one of the most established within medical research. The method is based on the Raman effect, which was discovered in 1928 by C.V. Raman and K.S. Krishnan [24]. When subjected to a high intensity laser source, a molecule will scatter the incident light. Elastic photon scattering, also called Rayleigh scattering, occurs when the wavelength of the incident photon is the same as the scattered photon after interacting with the tissue. The Raman effect refers to a small percentage of photons (1:10 million) that undergo inelastic scattering and change wavelength by either absorbing from or losing energy to the tissue. The resulting Raman spectrum serves as a unique chemical fingerprint of the tissue [24].

In biological tissues, the vibrational modes of different molecules in the tissue in combination with the composition of nucleic acids, proteins, and lipids, give the tissue a specific spectrum of Raman scattering [25]. Utilizing sophisticated spectroscopic techniques together with laser excitation, the Raman scattering can be detected and converted into spectral data for the material observed [26]. 

Raman spectroscopy has proven to be a well-suited technique for characterization of biological tissues such as tumors [16,27]. It is non-invasive, simple to operate and requires minimal sample preparation compared to previous techniques, e.g., 5-ALA [13]. Within brain tumor surgery, the Raman-based methods can be used for improving diagnostics of brain tumor biopsies [28], in Vivo tumor detection [29], molecular classification [30] and intraoperative histopathologic characterization [31]. The Raman-based imaging approaches that are most broadly used in neurosurgery include: stimulated Raman spectroscopy (SRS), stimulated Raman histology (SRH) and coherent anti-Stokes Raman scattering microscopy (CARS) [32]. 

There are still obstacles to overcome. Since only one in ten million photons is Raman scattered, the technique faces challenges in detecting low sample concentrations, especially in the context of competing phenomena such as elastic scattering and absorption effects. Besides the intrinsically weak Raman signal, other limitations include limited imaging depth, a limited field of view as well as slow imaging speed [33]. Hence, measuring the Raman effect requires sophisticated optical technologies to be compatible with the neurosurgical workflow. Pioneering research is underway to develop such technologies, with recent additions such as handheld fiber-optic Raman probes which can distinguish cancer from healthy brain, with high sensitivity and specificity [34]. These probes operate in real-time, requiring <0.2 s to measure spectra and have shown to be easily integrated into the neurosurgical workflow. 

Raman spectroscopy can thus provide diagnostic information in a shorter time compared to histopathology, the current gold standard for tissue diagnostics. RS data is acquired and processed within seconds, while intrasurgical histopathology takes tens of minutes [35]. 

### 1.2. Hyperspectral Imaging 

Multispectral and hyperspectral imaging are spectral-based imaging modalities that acquire data in almost contiguous narrow spectral bands. They allow the capture and interpretation of wavelengths and color bands beyond our physiological capability (which is a narrow range consisting of three color bands—red (620–750 nm), green (495–570 nm) and blue (450–495 nm). The difference between multispectral and hyperspectral lies in the count and width (nm) of the scanned wavelength range: hyperspectral includes bands with narrow widths (10–20 nm) and up to hundreds or thousands of them, whereas multispectral includes three to ten wider bands. From now on, we will refer to them both as “hyperspectral imaging”. 

The spatial and spectral data extracted in hyperspectral imaging is presented as a three dimensional (3D) hyperspectral data cube, which forms a set of 2D images [36]. Each pixel in the 2D plane provides a unique spectral signature reflecting the chemical composition of that particular pixel [37]. These 3D cubes serve as input for different computational techniques for visualizing and interpreting the data to differentiate different tissues [19]. With the recent progress in machine learning algorithms, these technologies are raising interest for various applications within the neurosurgical field [19,38]. 

The primary benefit of this technology is that it only uses white light to acquire the wavelengths of interest, thereby detecting spectral information in a non-invasive, non-ionizing way and without any physical contact [19]. The ability to obtain real-time hyperspectral images is crucial and suits intraoperative surgical use. In addition to differentiating between healthy and tumor brain tissue [39,40], hyperspectral imaging has been used to monitor intra-operative tissue oxygenation [41] as well as create real-time anatomical maps of the surgical space in other organs [42]. Despite these capabilities, the technique is not in regular surgical use yet due to lagging hardware development. More research is needed to miniaturize hyperspectral sensors and develop adequate real-time imaging models. 

### 1.3. Optical Coherence Tomography 

Optical Coherence Tomography is an established optical imaging technique that has been implemented in clinical practice within medical specialties including cardiology [43], dermatology [44], and neurosurgery [45]. One of the first areas of application was ophthalmology, where it was first used for in vivo retinal imaging by Fercher et al. [46]. 

OCT is a non-invasive, label-free, and cost-effective technique, capable of providing high-resolution, continuous, and three-dimensional tissue imaging [18]. It is based on utilization of broad-bandwidth light sources and interferometry with a low coherence length. The emitted light is coupled into an interferometer, a device that extracts information from interference. There are two light arms in the system, a sample arm, and a reference arm. The sample arm emits light toward the sample of interest, usually combined with an objective lens to focus the light, and the reference arm towards a mirror. Backscattered light from the sample and light from the reference are combined to generate an interference pattern that is detected by a detector. Two-dimensional or three-dimensional models are then reconstructed by scanning through the sample surfaces [47]. 

OCT can have image resolutions of 1–10 µm in all analyzed dimensions and is optimal for transparent or semi-transparent objects of limited depth, making it well suited for imaging biological tissue [48]. There is growing interest for OCT in neurosurgery as it can deliver continuous feedback to the surgeon with imaging depth (1.5–3 mm) that is comparable to resection depth for cancer-infiltrated brain tissue [49]. Currently the two main types of instruments used are handled probes and microscope-integrated OCT systems [50,51]. These handled probes demonstrate good resolution and field of view; while the microscope OCT-systems developed so far are very flexible, allowing an OCT-Camera to be attached to many different surgical microscopes.

### 1.4. Diffuse Reflectance Spectroscopy 

Diffuse Reflectance Spectroscopy is an optical technology that is based on elastic scattering of light, as opposed to Raman, which is based on inelastic scattering. Elastic scattering occurs more frequently than inelastic scattering to a factor of roughly 10:1 [24]. This allows DRS to collect scattering data much faster than Raman spectroscopy, providing faster reading times intraoperatively. In DRS, the optical fiber probe collects light originally emitted by the illumination fiber after it is partially scattered back by the tissue. The partial scatter is a result of absorption, reflection, transmission, and scattering. The elastic scattering can be used for precise optical characterization of tissues [52]. The molecular composition of a tissue determines the light absorption and thus the DRS-fingerprint of the specific tissue. Light absorption is mainly related to the types and concentration of endogenous chromophores present within tissues (e.g., fat, water, collagen, hemoglobin, beta-carotene, melanin, myoglobin) [53].

All the four mentioned technologies are non-invasive and capable of providing real-time feedback during intraoperative setups. HIS, RS and DRS technologies can provide information about the bulk properties of the tissue, such as oxygenation and blood volume but also by detecting spectroscopic patterns unique to the studied tumors. RS typically works at a smaller scale (subcellular) as opposed to HIS and DRS. OCT offers an outstanding axial spatial resolution (1.5–3 mm), thereby approaching the spatial resolution of conventional histopathology. 

In summary, given the ongoing technological advancements within the field of neurosurgery [54,55,56], the novelty of these techniques warrants a closer and systematic scrutiny to determine their potential added value. In an attempt to provide alternative technologies as a complement to current neuronavigational image-based and fluorescence-based solutions, this study aimed to investigate the use of four intrinsic optical imaging techniques, which already have evolved from pre-clinical settings to clinical applications in brain tumor surgery. We believe that these technologies have the highest potential for incorporation in modern decision support tools for neurosurgery. The aim of this systematic review is hence to compile evidence for the use of each of these techniques in brain surgery and to investigate their benefit in correctly distinguishing brain tumor tissue from healthy brain tissue. As a primary outcome, the synthesis will be focusing on sensitivity, specificity and accuracy data, as well as indicating the knowledge gaps that require further investigation and understanding.

## 2. Materials and Methods

This systematic review protocol was reported in accordance with the Preferred Reporting Items for Systematic Reviews and Meta-Analyses Protocol (PRISMA-P) checklist from 2015 (Supplementary file S1, Table S1). For transparency, the protocol was registered in the International prospective register of systematic reviews (PROSPERO), an established and widely used platform [54,57,58,59] (ID: CRD42022335836, Date of registration: 7 June 2022). A summary of the search strategy has been provided (Supplementary file S1, Table S2).

### 2.1. Type of Studies 

All original articles published in the English language were eligible for inclusion. Reviews, editorials, and letters to the editor were excluded. To maintain relevancy and adhere to recent knowledge, studies that were published prior to January 1990 were also excluded.

### 2.2. Type of Population

Studies performed on human brain tumor tissue, either in vivo or ex vivo were included. Tumor types include any intracranial tumors, ranging from primary benign or malignant tumors to brain metastases. For inclusion, at least five samples had to be included in the study’s analysis.

### 2.3. Types of Intervention

Methods involving exogenous fluorescent molecules or histopathological dyeing were excluded. Only studies investigating optical methods relying on the intrinsic properties of the tissue were considered. The optical methods of interest were as follows: (1) Hyperspectral Imaging, (2) Raman Spectroscopy, (3) Optical Coherence Tomography, and (4) Diffuse Reflectance Spectroscopy. These methods were chosen based on a review of the literature that aimed to yield the most interesting techniques within the field, as well as from consultation of experts in the field. 

### 2.4. Types of Comparators 

The studies that were included used any of the following comparators: healthy brain control tissue or any other tumor type.

### 2.5. Types of Outcome Measures 

The primary outcome of interest consisted of quantitative precision measures. These measures included sensitivity, specificity, and accuracy. All studies that did not report at least one of these quantitative precision measures were excluded.

### 2.6. Sources of Information and Search Strategy 

Searches were performed on Medline (Ovid), Web of Science, and Embase using a combination of the keywords, “brain” and “tumor”, with related entry terms specifically relating to each of the optical methods investigated (ex. “brain” “tumor” “diffuse reflectance spectroscopy”).

### 2.7. Data Selection

The screening of articles was performed on the Rayyan software [54,57,58,59,60,61] by two independent and blinded reviewers (MA) and (GB). In the first stage of the screening process, titles and abstracts were screened and irrelevant articles were extracted. In the second stage, the full texts of the remaining articles were retained. The retained full texts were assessed for inclusion based on the mentioned eligibility criteria. Any discrepancy in the article selection process was resolved via discussion and unanimous decision. 

### 2.8. Data Extraction

A data extraction template was designed to maintain a consistent extraction of data throughout the review (Supplementary file S1, Table S3).

### 2.9. Synthesis of Data 

As the field of optical methods within brain tumor surgery is still unexplored, methodological heterogeneity in study design, data analysis, statistical methods, tumor types and diagnostic algorithms among the studies included was observed. Hence, it was not applicable to conduct a meta-analysis. Instead, a qualitative synthesis of the results of the included studies was performed, with a focus on study characteristics as well as outcomes in terms of sensitivity, specificity, and accuracy.

## 3. Results

**Figure 1:**
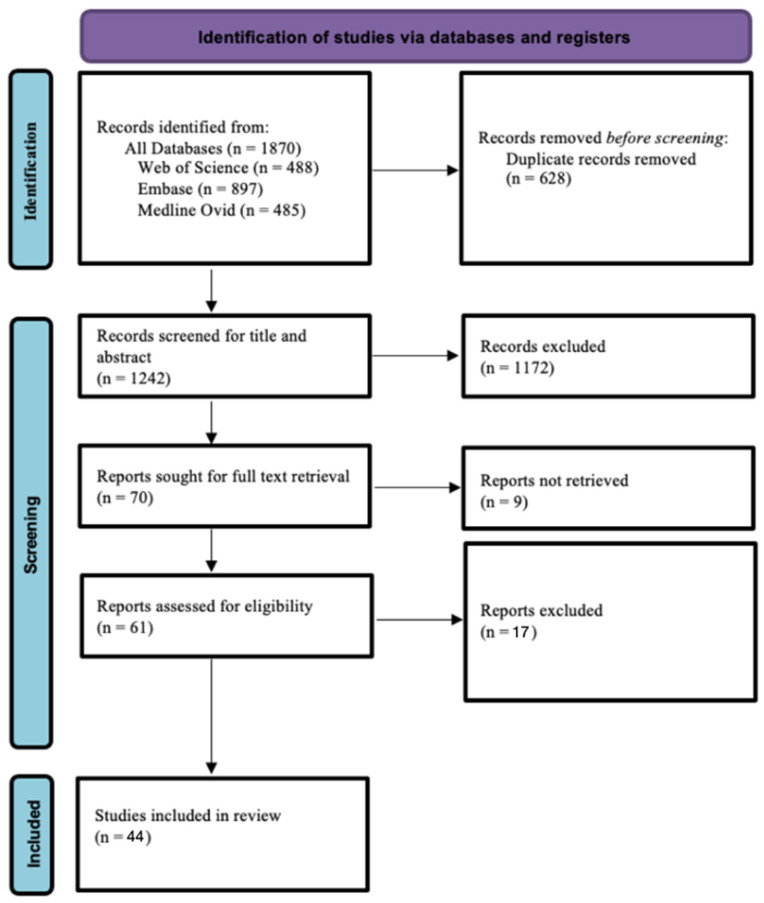
PRISMA flow chart diagram.

The initial searches in Web of Science, Medline (Ovid), and Embase resulted in 488, 485, and 897 records, respectively. In total, 1870 records were obtained. Following the screening process, 44 articles were included in this systematic review (Figure 1).

### 3.1. Raman Spectroscopy

#### 3.1.1. Study Characteristics

Raman spectroscopy (RS) was the most used with 31 studies (70.5%) investigating its use (Supplementary file S1, Table S4). Most of these studies were based in the US. Twenty-seven studies involved ex vivo tissues while only four performed optical analysis on in vivo tissues. The numbers of patients/samples ranged from 8 to 278. The studies applied a variety of diagnostic algorithms for classification of spectral data, with principal components analysis being the most mentioned, followed by support vector machines and linear discriminant analysis. Glial tumors, including glioblastoma multiforme, astrocytoma, and oligodendrogliomas were the most investigated, followed by meningiomas, and brain metastases. 

#### 3.1.2. Study Outcomes 

A large heterogeneity in terms of diagnostic accuracy was detected among the studies (Table 1). Overall, the reported sensitivity ranged from 24% (Stables et al. [62]) to 100% (Livermore et al. [63], Sun et al. [64], Leslie et al. [65]), the specificity from 19% (Stables et al. [62]) to 100% (Aguiar et al. [66], Sun et al. [64], Livermore et al. [59], Leslie et al. [65]), and accuracy from 54% (Stables et al. [62]) to 100% (Koljenovic et al. [67,68]).

### 3.2. Hyperspectral Imaging (HIS)

#### 3.2.1. Study Characteristics

Five studies investigating HIS were included (Supplementary file S1, Table S5). Four of these studies involved the same research group based in Spain. Four studies used the same Hyperspectral Imaging system (Hyperspec^®^VNIR A-Series, Headwall Photonics Inc., Fitchburg, MA, USA). While four of the studies analyzed images in an in vivo setting, one study analyzed ex vivo tissue samples. All of the studies involved the analysis of normal brain tissue as a comparator. Six different diagnostic algorithms were used across studies, all yielding different results. Urbanos et al. [90] and Ortega et al. [91] both used the conventional support vector machine (SVM), random forest (RF), convolutional neural networks (CNN), and artificial neural networks (ANN). In two of their articles, Fabelo et al. focused on 1D-CNN and 2D-CNN methods [92,93], while Manni et al. [94], in their study, proposed a 2D-3D-CNN hybrid model. 

#### 3.2.2. Study Outcomes

Sensitivity values ranging from 32% (Urbanos et al. [90]) to 98% (Ortega et al. [91]) and specificity values from 71% (Ortega et al. [91]) to 100% (Fabelo et al. [92], Fabelo et al. [93]) were reported among the included studies (Table 2). The best performing diagnostic algorithms in terms of accuracy were RF (Urbanos et al. [90] and Ortega et al. [91]) as well as 1D-DNN (Fabelo et al. [92,93]).

### 3.3. Optical Coherence Tomography (OCT)

#### 3.3.1. Study characteristics

A total of four studies on OCT were included in this review (Supplementary file S1, Table S6). These studies were based in the USA, Russia, and Germany. Möller et al. [95] and Kut et al. [96] studied ex vivo human samples, Juarez-Chambi et al. [97] focused on in vivo samples, while Yashin et al. studied both ex vivo and in vivo tissues [98]. The largest patient population was seen in Yashin et al. [98]. The tumors investigated in these articles included metastases, astrocytomas, low-grade gliomas, and glioblastoma multiforme. Various OCT-setups were used, including SD-OCT, TD-OCT, and cross polarization OCT (CP-OCT). All studies included the analysis of normal brain tissue as a control.

#### 3.3.2. Study Outcomes

The study outcomes are summarized Table 3. In the study by Yashin et al. [98], the combination of both co-polarization and cross-polarization setups yielded a higher accuracy (87–88%) when compared to the separate accuracy of each of the two systems. Juarez-Chambi et al. [97] reported the precision of their system in distinguishing low-grade from high-grade tumors, achieving sensitivity and specificity of 91% and 83%, respectively (Table 3). The highest accuracy in the study by Möller et al. [95] (99%), was achieved when distinguishing healthy from necrotic tissue. 

Overall sensitivity values for the OCT studies ranged from 80% (Yashin et al. [98]) to 100% (Kut et al. [96]), specificity values ranged from 67% (Yashin et al. [98]) to 100% (Kut et al. [96]) and accuracy values from 82% (Yashin et al. [98]) to 100% (Kut et al. [96]). 

### 3.4. Diffuse Reflectance Spectroscopy (DRS)

#### 3.4.1. Study Characteristics

Four DRS studies were included in this review (Supplementary file S1, Table S7). Three of these studies originated from the USA and one from Canada. Two of the studies shared the same first author (Lin et al. [99,100]). All articles used a system which employed a hand-held fiber optic probe. 

Except for the study by Du Le et al. [101], where ex vivo samples were analyzed, all other studies involved in vivo samples. The number of patients ranged from 7 to 35 with the largest sample being represented in the study by Majumder et al. [102] The tumor tissues analyzed included several types of gliomas and metastases. In the studies by Lin et al. [99,100], multiple pediatric brain tumors were studied. 

#### 3.4.2. Study Outcomes 

The study outcomes are summarized in Table 4. In the study by Du Le et al. [101], sensitivity and specificity of 100% and 90% were achieved by the DRS-based system when attempting to differentiate GBM from low-grade gliomas (Table 4). Majumder et al. [102] and Lin et al. [100] (2) studied the precision in detecting tumor margins from healthy brain tissue. Majumder et al. [102] applied two different diagnostic algorithms: maximum representation and discrimination feature-sparse multinominal logistic regression (MRDF-SMLR) and nearest-mean classifier (NMC). They reached accuracies of 94% in detecting tumor margins in MRDF-SMLR and 53% with NMC when running their validation sets.

Moreover, Lin et al. [100] (2) used both a single-step and a two-step discrimination algorithm in conjunction with the DRS system. When delineating tumor margins from a background of healthy brain tissue, the single-step discrimination algorithm yielded a sensitivity of 78% and a specificity of 76%, whereas the two-step discrimination algorithm achieved a slightly higher sensitivity: 89% and 76% for sensitivity and specificity, respectively. 

Overall, the sensitivity of the DRS studies ranged from 75% in the second study by Lin et al. [100] to 100% in the study by Du Le et al. [101], while specificity ranged from 66% in the first study Lin et al. [99] to 90% in the one by Du Le et al. [101] Accuracy was only reported in the study by Majumder et al. [102] ranging from 42% to 100%, depending on the comparators considered.

## 4. Discussion

In this systematic review, the literature has been explored to search for data on the sensitivity, specificity, and accuracy of detecting human brain tumor tissue of four emerging optical methods (RS, HIS, OCT and DRS). The main aim was to identify studies that reported sensitivity, specificity, and accuracy on the precision of the optical methods. From these results it can be concluded that RS is clearly the most explored of the four technologies, whereas HIS, OCT and DRS are less examined. Moreover, studies that report significant levels of precision for distinguishing brain tumor from healthy tissue, using technologies based on these optical methods have been presented.

### 4.1. Raman Spectroscopy 

Stables et al. [62] reported the lowest ratings in all three measures of sensitivity, specificity, and accuracy. This study used a method that generated and analyzed sound waves based on Raman spectra to distinguish between healthy and brain tissue. This alternative approach is worth exploring further as it can provide real-time auditory feedback to the neurosurgeon, without distracting their visual attention. However, improvement is needed regarding sensitivity and specificity. Eventual challenges including noise interference and user training must also be tackled before clinical implementation. 

Studies reporting higher sensitivity and specificity measures used the conventional optical approach to decipher RS data (Jermyn et al. [81,82,84], Livermore et al. [63]). Applying their RS model on brain biopsies taken from patients who underwent supramaximal glioma resections, Livermore et al. [63] could report that most of these biopsies contained infiltrating tumor cells despite supramaximal resections. They further compared the performance of RS to 5-ALA in these margin biopsies, where 5-ALA-induced fluorescence failed to detect most of the infiltrating tumor cells. These results highlight the better precision RS provides and how it can be utilized to guide the surgeon in the critical tumor margin region.

One of the latest studies (Baria et al. [35]) compared individual spectroscopic techniques with a multimodal approach (combining fluorescence, RS and DRS) to differentiate between brain tumor cells and dysplastic cells, reporting a higher accuracy with the multimodal approach compared to RS alone. Multimodal approaches can provide a more comprehensive evaluation of the examined tissue by exploiting different biological features, making them a promising avenue for future exploration. Another extensively researched area in the field is the use of machine learning. RS data is being used to train robust machine learning algorithms that can provide rapid and expert-level intraoperative diagnosis of brain tumors, practically replacing a traditional pathology laboratory (Hollon et al. [75,80]). 

A main reason for all this success is the many new tumor biomarkers being identified using RS data. The heterogeneity found in brain cancer requires a wide range of molecular fingerprints to distinguish different tumor types. In the studies included here Stables et al. [62] reported increased choline content in glioblastoma tissue while tryptophan and 2-hydroxyglutarate (2HG) activity was correlated with gliomas (Stables et. al. [62], Sun et al. [64]). In order to discriminate between low- and high-grade gliomas, RS markers for low-grade glioma include proline/tyrosine and choline/cholesterol, while phenylalanine and tryptophan are linked to high-grade gliomas [103]. Medulloblastoma has been shown to demonstrate a rise in the lipid-to-protein ratio when compared with normal brain tissue [104]. RS data have also demonstrated a conformational change from α-helix to β-sheets during tumor progression [104]. Hence, beyond guiding intraoperative tumor resection, RS is also contributing significantly to the field of neuro-oncology by detecting new tumor biomarkers. 

### 4.2. Hyperspectral Imaging

Urbanos et al. [90] and Ortega et al. [91] reported contrasting differences in sensitivity values despite training their models with similar ML algorithms and on the same tumor type, glioblastoma. Both studies utilized supervised ML algorithms including SVM, RF, CNN and ANNs. However, Urbanos et al. [90] acquired their data from in vivo images obtained during surgery while Ortega et al. [91] used ex vivo pre-diagnosed Glioblastoma pathology slides. 

Urbanos et al. [90] reported much lower sensitivity as the in vivo data showed high similarities between healthy and tumor glioblastoma tissue. This can be expected in the case of infiltrative tumors like glioblastoma where the healthy-labelled tissue is not always completely healthy. On the other hand, the same algorithms reported much higher sensitivity when trained on ex vivo tissue (Ortega et al. [91]). This variation in results is likely to be multifactorial, depending on tissue state, tissue labelling, and the ML model used, as studies combining in vivo glioblastoma data with other ML algorithms have reported higher sensitivity measures (Manni et al. [94], Fabelo et al. [92,93]). 

Both in vivo and ex vivo models serve important roles in neuro-oncology where in vivo models can guide brain tumor resection in real-time while ex vivo models are a useful help for the neuropathologist. Another main reason for result variation across these studies is the small number of included patients as machine learning algorithms require considerable amount of data to be specific. The current lack of robust discrimination between healthy and tumor tissue therefore limits the generalization of these studies for clinical use.

### 4.3. Optical Coherence Tomography 

Earlier work done in the OCT field has relied on estimating the tissue optical attenuation coefficient form the OCT signal to distinguish between cancerous and healthy tissue, with Kut et al. [96] showcasing impressive diagnostic precision in ex vivo tissue. For patients with higher-grade tumors, the achieved sensitivity/specificity reached 92%/100%, while for low-grade tumors, sensitivity/specificity values were 100%/80% (Kut et al. [96]). However, this approach requires sacrificing spatial resolution to boost signal quality. To overcome this, alternative approaches have been used in recent years. 

One such approach was conducted by Juarez-Chambi et al. [97] where they used a novel AI-assisted computational pipeline on in vivo glioma tissue to overcome the low spatial resolution limiting previous work. Their approach was able to differentiate between low-grade and high-grade gliomas with high precision. 

Another novel approach was conducted by Yashin et al. [98] where they used a cross-polarization OCT (PS-OCT) approach which can detect both the light scattering and the polarization properties of the tissue, and thereby provide tissue-specific contrast and better visualization of structures like myelinated nerve fibers [105]. The study achieved high diagnostic accuracy (87–88%) for differentiating white matter and tumor tissue. The experiments performed in vivo by Yashin et al. [98] also included a more heterogenous mix including astrocytomas, glioblastomas and breast cancer metastasis and showed overall that cancerous tissue is characterized by a lower optical attenuation rate when compared to healthy white matter, findings also reported by Kut et al. [96]. 

Another major benefit for OCT technologies is their high processing speed, with newer approaches acquiring high-resolution images under 1 s (Juarez-Chambi et al. [97]). This time efficacy combined with high resolution makes them suitable for real-time in situ detection of brain cancer. 

### 4.4. Diffuse Reflectance Spectroscopy 

Lin et al. [99] (1) performed their study on a pediatric population and included major pediatric tumor types including pilocytic astrocytoma, ganglioglioma, and medulloblastoma, making the analyzed tumor tissue much more heterogeneous compared to other studies in the optical field which focus mainly on gliomas and glioblastomas. Their intraoperative setup with a handheld probe was able to distinguish between healthy and cancerous tissue, reporting that diffuse reflectance intensities between 600 and 800 nm were most effective for discrimination. However, the spectral analysis and classification methods used here did not include the entire spectral data, therefore not capturing the complete biological variation present in the tissue. 

Further work in the field has been able to improve on this setup with Du Le et al. [101] combining fluorescence spectroscopy and DRS to differentiate between low-grade gliomas and glioblastoma multiforme, with a reported 100% sensitivity in ex vivo tissue. They reported the distinguishing feature to be higher scattering and absorption coefficients in glioblastoma multiforme compared to low-grade gliomas. 

Elmi-Terander et al. studied differentiation between low-grade gliomas and healthy brain tissue specifically [22]. Classification using random forest yielded a sensitivity of 82.0% and a specificity of 82.7% for the detection of low-grade gliomas. Their method involved a fitting model for estimating biological constituents in addition to scattering and absorption coefficients. 

### 4.5. Applications

Optical methods are emerging as a new and innovative way for intraoperative tumor detection [20,22]. Within the field of brain tumor surgery, there is a demand for improved ways of detecting tumor tissue intraoperatively so that a maximal extent of resection can be achieved. Improved extent of resection can result in decreased tumor recurrence which in turn can result in decreased morbidity and mortality [1]. Furthermore, optical methods have the potential to improve the precision and diagnostic rapidity of brain tumor biopsies, making brain biopsies less harmful [106]. These optical methods are also under investigation for a wider range of implementations in neurosurgery including studying cerebrovascular plaque composition [107], enhancing precision in functional neurosurgery, intraoperative microcirculation measurements [108] and diagnostics. Optical methods have the potential to revolutionize surgery and many more applications are yet to be explored. 

However, for the optical methods to advance from an experimental stage to clinical intraoperative practice, it is essential that studies are performed on a wide variety of tumor types, to capture data on the precision of various system-setups, diagnostic algorithms, and spectral signatures of tissues. This review showed that RS had the largest number of studies and the most inclusive range of tumor types, hence being the most mature of the optical methods. As for HIS and OCT the range of tumor type representation was limited. 

Most of the research conducted in the field suffers from tumor homogeneity. Overall, glioblastoma multiforme was the most investigated tumor type. Breakthroughs in the surgical treatment of this highly malignant and infiltrative tumor [109] would have a major impact on the field, leading to an expected high research incentive. Surprisingly, a range of studies also investigated meningiomas, which are common extra-axial tumors with no infiltrative features. Adhering to relevant research questions within the field is key to highlighting the utility and potential benefit of a novel technology. Hence, future studies ought to focus more on invasive or intraparenchymal tumors that are difficult to distinguish from normal surrounding brain tissue. 

Another major drawback in most studies is the small number of tissue samples included. This needs to change going forward particularly as AI is progressively utilized for outcome prediction. Future work needs to address these issues in a constructive way. For example, while training models on heterogeneous tumor types is very important, studies training their models on many different tumor types without increasing the sample size, risk limiting the generalizability of the results. 

### 4.6. In Vivo/Ex Vivo Setups

There are two ways for the discussed optical systems to be used intraoperatively: (1) through a hand-held fiber optic probe that is contact-based and analyzes the tissue in vivo, and (2) through a stationary device that is set up near the operating table and that analyzes ex vivo tissue with instant feedback to the surgeon. The latter essentially replaces a pathologist, cutting down on the logistical hurdles of preserved tissue transportation to a pathology lab, and minimizing the intraoperative waiting time for histopathological analysis. 

In the case of Raman-based technologies, studies by Jermyn et al. [81,82,84] used a hand-held fiber optic probe to perform a contact-based spectral analysis of brain tissue in vivo while Hollon et al. [75] (1) used a “stimulated Raman histology” system that classified images with convolutional neural networks. Similar in vivo/ex vivo setups exist for DRS and OCT. 

As most research performed so far has been ex vivo, more in vivo data is needed. For more in vivo contact-probe-based studies to be performed, a high ethical standard must be upheld; and for this, more evidence of the safety, precision and reliability of the technologies is needed. Overcoming challenges in future in vivo setups is therefore crucial, including the standardization of probe stability, addressing variations in probe contact pressure that may impact spectral measurements, minimizing signal loss from the probe fiber, and overcoming reduced performance caused by the presence of blood in the measurement field.

Another main constraint linked to the regular clinical application of these technologies is the necessity for a well-established illumination setup to minimize interference from external light sources in the recorded signal. Optical filters and other novel engineering solutions can be used to limit the effect of these sources when designing future operating rooms.

### 4.7. Sensitivity and Specificity

It is important to acknowledge that the sensitivity and specificity of these optic methods for detecting tumor tissue needs to be adapted to the specific surgical procedure for which they will be used. For example, when operating in proximity to eloquent areas of the brain, a specific (low false-positive rate) system that reserves the surrounding healthy tissue is desired. Similarly, in the case of brain tumor biopsies, systems of high specificity are desired, as the aim is to specifically sample the tumor tissue. On the other hand, operating on highly invasive tumors, where the loss of surrounding brain tissue has a negligible impact, a high sensitivity (low false negative rate) would be desired.

### 4.8. Limitations

First, our systematic review focused on four of the most popular optical methods, leaving out less established tools and limiting the generalizability of the findings to the whole field of optics. In addition, it is undeniable that the field of optical methods in neurosurgery is still in its initial stages. For that reason, the external validity of the findings highlighted in this review is limited. Finally, due to the methodological heterogeneity between studies, a meta-analysis could not be performed.

### 4.9. Future Perspectives

Despite huge technical advances within the field of neurosurgery regarding navigation and tissue identification, several challenges remain. To date, no widely used tools to enhance surgeon’s vision and provide immediate tissue diagnostic are available. The naked eye, even equipped with the surgical microscope, cannot differentiate between diffuse growing tumors and healthy brain tissue. Intraoperative pathological diagnostics is slow and valuable intraoperative time is lost for that purpose. Photosensitive drugs and contrast agents are not without side effects and limitations. The phenomenon of brain shift adds to the difficulty of navigation and tissue recognition. We believe that incorporation of intrinsic optical technologies could provide parts of the required solutions. However, these technologies generate huge amounts of data requiring the ability to handle and interpret big data simultaneously with the surgical procedure. The future research efforts will therefore require collaboration between neurosurgeons, computer scientists, experts in optical technologies and navigation. One such initiative is the project STRATUM [110], a 3D decision support tool for brain tumor surgery within the European Union Horizon Program. However, more international and interdisciplinary efforts are required to successfully progress this field.

## 5. Conclusions

In this systematic review, the characteristics of the optical systems, tumor types and the outcomes in terms of sensitivity, specificity and accuracy have been summarized. The results show that there is evidence for all four optical methods being promising for identification of brain tumors and healthy tissue. Raman spectroscopy is currently the most explored method. More studies, however, are needed for the technologies to move from an experimental stage to an intraoperative clinical setting.

## Figures and Tables

**Table 1:** Study outcomes. Tables listing precision outcomes in terms of sensitivity, specificity, and accuracy for RS studies.

Study	Classifier	Sensitivity %		Specificity %		Accuracy %		
Riva et al. [69]	Normal vs. glioma	N/A		N/A		RF	GB	
						80	83	
Sciortino et al. [70]	IDH-mut vs. IDH-wildtype	N/A		N/A		RBF-SVM	XGB	
						87	85	
Kopec et al. [71]	MET	95		86		N/A		
	GS	95		50				
	AOD III	100		99				
	MEN II	90		80				
	MT	90		92				
	PT	96		62				
	NF	90		73				
Jelke et al. [72]		Cross-validated	Hold-out external validation	Cross-validated	Hold-out external validation	N/A		
	Meningioma vs. dura	96	100	95	94			
Pekmezci et al. [73]	Raman vs. H&E Raman vs. IHC Assessment of WHO-grade	Raman vs. H&E = 86 Raman vs. IHC = 88		Raman vs. H&E = 86 Raman vs. IHC = 81		WHO II = 81 (by specimen) WHO II-IV = 81 (by specimen) WHO II = 100 (by patient) WHO II-IV = 73 (by patient)		
Aguiar et al. [66]		LDA	PLS-DA	LDA	PLS-DA	LDA	PLS-DA	
	G vs. Cerebellum	96	98	91	97	93	98	
	MEN vs. Meninges	99	99	100	100	99	99	
	Combined						94	
Livermore et al. [63]	Tumor	96		99		99		
	Normal brain	99		96		99		
	Raman (overall)	100		100		100		
Bury et al. 1 [74]		PCA-QDA		PCA-QDA		PCA-QDA		
	Normal vs. tumor (MEN and G)	99		42		94		
Hollon et al. 1 [75]	Raman	N/A		N/A		95		
	Pathologist					94		
Bovenkamp et al. [76]	Pathological tissue vs. pituitary gland tissue	N/A		N/A				
	Corticotroph					84		
	Gonadotroph					88		
	Somatotroph					99		
	Plurihormonal					91		
	Null cell					91		
	Pituitary gland					95		
	Periosteal layer					100		
Sun et al. [64]	wavenmr/cm^−1^	350–2000	1200–1600	350–2000	1200–1600	350–2000	1200–1600	
	Normal vs. Tumor							
	PLS	100	100	100	100	100	100	
	SVM	91	96	100	100	96	98	
	ANN	100	100	91	100	94	100	
Morais et al. [77]		PCA-LDA, SPA-QDA		PCA-LDA, SPA-QDA2		PCA-LDA, SPA-QDA		
		86		100		96		
Galli et al. [78]	Tumor vs. non-tumor Spectra	N/A		N/A		93		
	Biopsy					95		
Uckermann et al. [30]	IDH-mut vs. IDH-wt	N/A		N/A				
	Validation set					89		
	Training set					88		
Bury et al. 2 [79]	vs each other	N/A		N/A				
	Colon					69		
	Lung					69		
	Melanoma					72		
	Colon+lung vs. melanoma							
	Colon+Lung					85		
	Melanoma					75		
Hollon et al. 2 [80]	Normal vs. lesional tissue	N/A		N/A		94		
	Low-grade vs. high-grade					89		
Jermyn et al. 1 Integrated [81]		RS	RS+IFS	RS	RS+IFS	RS	RS+IFS	
	G vs. normal	90	100	97	94	93	98	
	MET vs. normal	97	100	84	92	90	96	
	combined vs. normal	92	100	90	93	91	97	
Stables et al. [62]	Normal	N/A		N/A		71		
	MET					87		
	GBM					54		
Jermyn et al. 2 [82]	Invasive cancer vs. normal	92		93		92		
Liu et al. [83]	Normal vs. Glioma	N/A		N/A		86		
	Glioma vs. Normal					89		
Jermyn et al. 3 [84]	Normal vs. all cancer	93		91		92		
Desroches et al. [29]	Vital (tumor and normal) vs. Necrosis	84		89		87		
Ji et al. [85]	Raman							
	H&E							
	Tumor infiltrated vs. non tumor	98		99		95		
	Glioma-only GAM	97		99		92		
	Leave-one-out cross-validation	87		88				
Kalkanis et al. [86]	Necrosis vs. normal vs. GBM Training set	N/A		N/A				
	Validation set					98		
						100		
Bergner et al. [87]	Carcinoma vs. normal brain vs. necrosis vs. “remaining”	N/A		N/A		Linear SVM	Radial SVM	PLS-DA
	1st level training data					95	97	88
	1st level independent data					95	93	94
	2nd level training data					100	100	97
	2nd level training data					80	89	70
Auner et al. [88]	Normal vs. MB vs. G	N/A		N/A				
	Training					95		
	Testing					89		
	Combined classification					97		
Leslie et al. [65]	A vs. normal							
	Low-grade EP vs. normal							
	Low-grade G vs. normal							
	OD vs. normal							
	Tissue level							
	Low-grade G vs. high-grade G	87		98				
	Low-grade EP vs. high-grade	92		99.7				
	Training	92		98				
	All	92		100		100		
	Testing	100		100				
	normal	91		87		97		
	G	100		96		96		
	MB					92		
	Differentiation							
	A					85		
	EP					89		
	OD					61		
	GG					75		
Koljenovic et al. 1 [67]	MEN vs. dura	N/A		N/A		LDA		
						100		
Koljenovic et al. 2 [68]	Necrotic vs. vital	N/A		N/A		LDA		
						100		
Zhou et al. [89]	Glioma vs. Normal	100		96.3		99.6		
Baria et al. [35]	Raman alone							
	Control vs. Tumor	92.6		93.3		82.5		
	Control vs. Dysplasia	76.6		80				
	Tumor vs. Dysplasia	66.7		77.7				
	Multimodal approach					87.5		
	Control vs. Tumor	91		100				
	Control vs. Dysplasia	86		100				
	Tumor vs. Dysplasia	89		86				

**Abbreviations:** N/A = Not applicable. **Tumors:** A = Astrocytoma; AA = Anaplastic astrocytoma; AE = Anaplastic ependymoma; AG = Astroganglioma; AOD = Anaplastic oligodendroglioma; E = Embryonal; EP = Ependymoma; G = Glioma; GBM = glioblastoma; GG = Ganglioglioma; GM = Germinoma; GS = Gliosarcoma; HB = Hemangioblastoma; M = Meningioma; MB = Medulloblastoma; Met = Metastasis; MT = Meningothelioma; N = Normal; NF = Neurofibroma; OA = Oligoastrocytoma; OD = Oligodendroglioma; PA = Pilocytic astrocytoma; PT = Pituitary. **Diagnostic algorithms:** ANN = Artificial Neural Network; Bt = Boosted trees; CNN = Convolutional Neural Network; DA = Discriminant analysis; DFA = Discriminant Function Analysis; DNN = Deep Neural Networks; GAM = Generalized additive model; Gb = Gradient Boosting; KCA = K-means Cluster Analysis; KNN = K- Nearest Neighbour classifier; kNN = kernel Neural Network; LDA = Linear Discriminant analysis; PC = Principal Components; PCA = Principal Component Analysis; PLS = Partial least squares; QDA = Quadrantic Discriminant Analysis; RBF = Radial Basis Function kernel; RF = Random Forrest; SPA = Successive Projections Algorithm; SVM = Support Vector Machine; and XGB = eXtreme Gradient Boosted trees.

**Table 2:** Study outcomes. Table listing precision outcomes in terms of sensitivity, specificity, and accuracy for HIS studies.

Study	Tissue	Sensitivity %			Specificity %			Accuracy %		
Urbanos et al. [90]		SVM (A)	RF (A)	CNN (A)	SVM (A)	RF (A)	CNN (A)	SVM (A)	RF(A)	CNN(A)
	Overall							75	95	92
	Healthy	90	98	98	72	95	89	81	97	94
	Tumor	32	86	63	91	99	99	80	97	94
	Dura	79	97	96	96	99	99	93	99	99
Manni et al. [94]		2D-3D CNN (hybrid)			2D-3D CNN (hybrid)			2D-3D CNN (hybrid) 80		
	Mean									
	Normal	76			87					
	Tumor	68			98					
	Vessel	74			92					
	Background	87			87					
Fabelo et al. 1 [92]		1D-DNN		2D-CNN	1D-DNN		2D-CNN	1D-DNN		2D-CNN
		88		76	100		100	94		88
Fabelo et al. 2 [93]		1D-DNN		2D-CNN	1D-DNN		2D-CNN	1D-DNN		2D-CNN
		88		76	100		100	95		90
Ortega et al. [91]		SVM	ANN	RF	SVM	ANN	RF	SVM	ANN	RF
	(Tumor vs. normal)									
	Self as control	96	98	96	97	98	97	96	98	96
	Others + self as control	86	92	94	79	91	92	83	92	93
	Others as control	76	75	73	71	77	79	76	78	69

**Abbreviations:** ANN = Artificial Neural Networks, CNN = Convolutional Neural networks, DNN = Deep Neural Networks, RF = Random Forrest, and SVM = Support Vector Machines.

**Table 3:** Study outcomes. Table listing precision outcomes in terms of sensitivity, specificity, and accuracy for OCT studies.

Study	Classifier	Sensitivity %	Specificity %	Accuracy %
Möller et al. [95]	Vital tumor vs. healthy	N/A	N/A	96
	Healthy vs. necrosis			99
	Tumorous (vital + necrosis) vs. healthy			97
Yashin et al. [98]	Visual assessment of pics ex vivo			
	co-polarization	89–93	67–73	83–84
	cross polarization	80–87,	75–89	82–83
	combined	82–85	92–94	87–88
Juare-Chambi et al. [97]	Threshold of 80% determined by Receiver Operating Characteristic (ROC) analysis:			N/A
	Low grade vs. non-cancerous	90%	81	
	High grade vs. non-cancerous	95%	82	
	Low grade vs. high grade	91%	83	
Kut et al. [96]	Using optical attenuation threshold of 5.5 mm^−1^			N/A
	High grade = 92%	92	100	
	Low grade = 100%	100	80	

Abbreviations: N/A = Not applicable.

**Table 4:** Study outcomes. Table listing precision outcomes in terms of sensitivity, specificity, and accuracy for DRS studies.

Study	Classifier	Sensitivity %	Specificity %	Accuracy %	
Du Le et al. [101]	GBM vs. LGG 650 nm Cut-off			N/A	
	20% DR	100	80		
	0.6 cm^−1^	92	80		
	10 cm^−1^ at	100	90		
Lin et al. 1 [99]	Tumor vs. normal	95	66	N/A	
Majumder et al. [102]	MRDF-SMLR	N/A	N/A		
	Training set				
	Tumor			96	
	Tumor margin			80	
	Normal			97	
	Validation set				
	Tumor			96	
	Tumor margin			94	
	Normal			100	
	NMC				
	Training set				
	Tumor			49	
	Tumor margin			78	
	Normal			52	
	Validation set				
	Tumor			42	
	Tumor margin			53	
	Normal			49	
Lin et al. 2 [100]	Discrimination algorithm	Single-step	Two-step	Single-step	Two-step
	Normal vs. infiltrative tumor margin	81	100	76	-
	Normal vs. primary tumors				
	Normal vs. secondary tumors	75	84	-	-
	Overall	83	-	-	-
		78	89	76	76

**Abbreviations:** N/A = Not applicable. Tumor: GBM = glioblastoma multiforme, LGG = low-grade glioma. Diagnostic algorithm: MRDF-SMLR = Maximum representation and discrimination feature-sparse multinominal logistic regression, and NMC = Nearest mean classifier.

## Data Availability

The excel file used to conduct this systematic review can be provided by the corresponding author upon reasonable demand.

## Supplementary Materials

The following supporting information can be downloaded at: https://www.mdpi.com/article/10.3390/jcm13092676/s1, Table S1: PRISMA checklist; Table S2: Search strategy, Table S3: Data extraction, Table S4: Characteristics of studies involving Raman Spectroscopy, Table S5: Table listing the characteristics of all included studies investigating HSI, Table S6: Table listing the characteristics of all included studies investigating OCT, Table S7: Table listing the characteristics of all included studies investigating DRS.
