# Supplementary material for: Optical Methods for Brain Tumor Detection: A Systematic Review
Source: J Clin Med. 2024 May 2;13(9):2676. doi: 10.3390/jcm13092676 (PMC11084501; doi:10.3390/jcm13092676)
Supplement: Supplementary file 1 [file jcm-13-02676-s001.zip › jcm-2983293-supplementary.pdf]

Supplementary file 1

Raman Spectroscopy

Of the intrinsic optical methods, Raman Spectroscopy is one of the most established within medical research. The method is based on the Raman effect, which was discovered in 1928 by C.V. Raman and K.S. Krishnan [1], and later awarded the Nobel Prize in Physics. The Raman effect refers to the 1:10 million portion of photons that undergo inelastic scattering following excitation of electrons in a material. In biological tissues, the vibrational modes of different molecules in the tissue in combination with the composition of nucleic acids, proteins, and lipids, give the tissue a unique spectra of Raman scattering [2]. Utilizing sophisticated spectroscopic techniques together with laser excitation, the Raman scattering can be detected and converted into spectral data for the material observed [3] .

Raman Spectroscopy has been broadly used in medicine, not least in neurosurgery, and the adaptations are rapidly expanding. In particular, it has proven to be a well-suited technique for characterization of biological tissues such as tumors [4,5]. Within brain tumor surgery, the Raman-based methods can be used for improving diagnostics of brain tumor biopsies [6], in vivo tumor detection [7], molecular classification [8] and intraoperative histopathologic characterization [9]. The Raman-based imaging methods that are most broadly discussed in neurosurgery include: Spontaneous Raman spectroscopy (SRS), coherent anti-Stokes Raman scattering microscopy (CARS) and stimulated Raman histology (SRH) [10].

Hyperspectral Imaging

Multispectral and hyperspectral imaging allow the capture and interpretation of wavelengths and color bands beyond our physiological capability (which is a narrow range of 380 to 780 nm, with three color bands). The difference between multispectral and hyperspectral lies in the count and width (nm) of the bands of color that they cover: hyperspectral includes bands with narrow widths (10-20 nm) and up to hundreds or thousands of them, whereas multispectral includes three to ten bands that are much wider. From now on, we will refer to them both as "hyperspectral imaging". When captured, each pixel of the hyperspectral image presents a more narrow and precise representation of the electromagnetic spectrum, and therefore gives us a more distinguished characterization of the tissue observed.

The data that is extracted in hyperspectral imaging is presented as a hyperspectral data cube. Spanning three dimensions, two dimensions (2D) of space (x and y) and a third dimension in spectra (wavelength), the data can be variable, and is able to distinguish various tissues and states of the tissues by their optical properties. The optical properties of the tissues themselves are determined by the molecular composition [11]. There are different computational techniques for visualizing and interpreting the data that is acquired through hyperspectral imaging. With the recent progress in acquisition and interpretation of data from these systems, they are attracting interest for various applications within the neurosurgical field [11].

Optical Coherence Tomography

Optical Coherence Tomography (OCT) is an established optical imaging technique that has been implemented in clinical practice within medical specialties including cardiology [12], dermatology [13], and neurosurgery [14]. One of the first and foremost areas of application was ophthalmology, where it was first used for in vivo retinal imaging by Fercher et al. [15].

OCT is based on utilization of broad-bandwidth light sources and interferometry with a low coherence length. The emitted light is coupled into an interferometer, a device that extracts information from interference. There are two light arms in the system, a sample arm and a reference arm. The sample arm emits light toward the sample of interest, usually combined with an objective lens to focus the light, and the reference arm towards a mirror. Backscattered light

from the sample and light from the reference are combined to generate an interference pattern that is detected by a detector. Two-dimensional or three-dimensional models are then reconstructed by scanning through the sample surfaces [16].
As OCT can have image resolutions of 1-10 µm in all analyzed dimensions and as it is optimal for transparent or semi-transparent objects of limited depth, it is well suited for imaging biological tissue [17].

Diffuse Reflectance Spectroscopy
Diffuse Reflectance Spectroscopy (DRS) is an optical technology that is based on properties of elastic scattering of light, as opposed to Raman's, which is based on inelastic scattering. In DRS, the optical fiber probe collects light originally emitted by the illumination fiber after it is partially scattered back by the tissue. The partial scatter is a result of absorption, reflection, and scattering. The elastic scattering can be used for precise optical characterization of tissues [18]. The molecular composition determines the results of the DRS-fingerprint of the specific tissue. The different degree and spectrum of light absorption in different tissues also play a role in the tissue-specific patterns obtained. Light absorption is mainly related to the types and concentration of endogenous chromophores present within tissues (e.g., hemoglobin, beta-carotene, melanin, myoglobin) [19].

Table S1
PRISMA-P 2015 Checklist

This checklist has been adapted for use with protocol submissions to *Systematic Reviews* from Table 3 in Moher D et al: preferred reporting items for systematic review and meta-analysis protocols (PRISMA-P) 2015 statement. *Systematic Reviews* 2015 4:1

**Section/topic**	**#**	**Checklist item**	**Information reported**		**Line number(s)**
			**Yes**	**No**	
**ADMINISTRATIVE INFORMATION**					
**Title**					
Identification	1a	Identify the report as a protocol of a systematic review	☒	☐	Line 1
Update	1b	If the protocol is for an update of a previous systematic review, identify as such	☐	☒	Not applicable
**Registration**	2	If registered, provide the name of the registry (e.g., PROSPERO) and registration number in the Abstract	☐	☒	Not applicable
**Authors**					
Contact	3a	Provide name, institutional affiliation, and e-mail address of all protocol authors; provide physical mailing address of corresponding author	☒	☐	Lines 460-470 and 484-488
Contributions	3b	Describe contributions of protocol authors and identify the guarantor of the review	☒	☐	Lines 472-483
**Amendments**	4	If the protocol represents an amendment of a previously completed or published protocol, identify as such and list changes; otherwise, state plan for documenting important protocol amendments	☐	☒	Not applicable
**Support**					
Sources	5a	Indicate sources of financial or other support for the review	☐	☒	Not applicable
Sponsor	5b	Provide name for the review funder and/or sponsor	☐	☒	Not applicable
Role of sponsor/funder	5c	Describe roles of funder(s), sponsor(s), and/or institution(s), if any, in developing the protocol	☐	☒	Not applicable
**INTRODUCTION**					
**Rationale**	6	Describe the rationale for the review in the context of what is already known	☒	☐	Lines 6-11 and 90-96

Section/topic	#	Checklist item	Information reported		Line number(s)
			Yes	No	
**Objectives**	7	Provide an explicit statement of the question(s) the review will address with reference to participants, interventions, comparators, and outcomes (PICO)	☒	☐	Lines 97-102
**METHODS**					
**Eligibility criteria**	8	Specify the study characteristics (e.g., PICO, study design, setting, time frame) and report characteristics (e.g., years considered, language, publication status) to be used as criteria for eligibility for the review	☒	☐	Lines 112-143
**Information sources**	9	Describe all intended information sources (e.g., electronic databases, contact with study authors, trial registers, or other grey literature sources) with planned dates of coverage	☒	☐	Lines 146-151
**Search strategy**	10	Present draft of search strategy to be used for at least one electronic database, including planned limits, such that it could be repeated	☒	☐	Line 150
STUDY RECORDS					
Data management	11a	Describe the mechanism(s) that will be used to manage records and data throughout the review	☒	☐	Lines 154-163 and 166-174
Selection process	11b	State the process that will be used for selecting studies (e.g., two independent reviewers) through each phase of the review (i.e., screening, eligibility, and inclusion in meta-analysis)	☒	☐	Lines 154-159
Data collection process	11c	Describe planned method of extracting data from reports (e.g., piloting forms, done independently, in duplicate), any processes for obtaining and confirming data from investigators	☒	☐	Lines 166-174
**Data items**	12	List and define all variables for which data will be sought (e.g., PICO items, funding sources), any pre-planned data assumptions and simplifications	☒	☐	Lines 167-172
**Outcomes and prioritization**	13	List and define all outcomes for which data will be sought, including	☒	☐	Lines 209-216

Section/topic	#	Checklist item	Information reported		Line number(s)
			Yes	No	
		prioritization of main and additional outcomes, with rationale			
**Risk of bias in individual studies**	14	Describe anticipated methods for assessing risk of bias of individual studies, including whether this will be done at the outcome or study level, or both; state how this information will be used in data synthesis	☒	☐	Lines 177-187
DATA					
**Synthesis**	15a	Describe criteria under which study data will be quantitatively synthesized	☐	☒	Not applicable
	15b	If data are appropriate for quantitative synthesis, describe planned summary measures, methods of handling data, and methods of combining data from studies, including any planned exploration of consistency (e.g., I^2, Kendall's tau)	☐	☒	Not applicable
	15c	Describe any proposed additional analyses (e.g., sensitivity or subgroup analyses, meta-regression)	☐	☒	Not applicable
	15d	If quantitative synthesis is not appropriate, describe the type of summary planned	☒	☐	Lines 218-228
**Meta-bias(es)**	16	Specify any planned assessment of meta-bias(es) (e.g., publication bias across studies, selective reporting within studies)	☒	☐	Lines 183-184
**Confidence in cumulative evidence**	17	Describe how the strength of the body of evidence will be assessed (e.g., GRADE)	☒	☐	Lines 189-204

Table S2. Summary table of search strategy. The final search results are marked using italics.

Source	Search		Results
**Web of Science**			
	#1	"Diffuse reflectance spectroscopy" OR "Raman spectroscopy" OR "optical coherence tomography" OR "multispectral imaging" OR "hyperspectral imaging" OR Spectral Analysis, Raman OR Spectroscopy, Near Infrared OR Tomography, Optical Coherence	272,092
	#2	Brain OR neurosurgery OR intracranial	1,528,093
	#3	Neoplasm OR neoplasma OR tumor OR tumors OR tumour OR tumours OR metastasis OT metastases OR glioma OR gliomas	2,201,636
	#4	*#1 AND #2 AND #3*	*488*
**Embase**			
	#1	"hyperspectral imaging"/exp AND ([article]/lim OR [article in press]/lim)	576
	#2	"multispectral imaging"/exp AND ([article]/lim OR [article in press]/lim)	228
	#3	"Diffuse reflectance spectroscopy"/exp AND ([article]/lim OR [article in press]/lim)	2,857
	#4	"Raman spectroscopy"/exp AND ([article]/lim OR [article in press]/lim)	41,416
	#5	"optical coherence tomography/exp AND ([article]/lim OR [article in press]/lim)	50,177
	#6	"Near Infrared OR Tomography"/exp AND ([article]/lim OR [article in press]/lim)	19,519
	#7	("hyperspectral imaging" OR "multispectral imaging" OR "diffuse reflectance spectroscopy" OR "raman spectroscopy" OR "optical coherence tomography" OR "near infrared spectroscopy:ab,kw,ti) AND ([article]/lim OR [article in press]/lim)	95,451
	#8	"neoplasm"/exp AND ([article]/lim OR [article in press]/lim)	3,333,893
	#9	neoplasm OR neoplasms OR tumor OR tumors OR tumour OR tumours OR metastasis OR metastases OR glioma OR gliomas:ab,kw,ti) AND ([article]/lim OR [article in press]/lim)	2,830,101
	#10	"brain"/exp AND ([article]/lim OR [article in press]/lim)	1,062,044
	#11	"neurosurgery"/exp AND ([article]/lim OR [article in press]/lim)	208,669
	#12	(brain OR neurosurgery OR intracranial:ab,kw,ti) AND ([article]/lim OR [article in press]/lim)	1,941,534
	#13	#1 OR #2 OR #3 OR #4 OR #5 OR #6 OR #7	123,717
	#14	#8 OR #9	3,898,772
	#15	#10 OR #11 OR #12	2,256,907
	#16	*#13 AND #14 AND #15*	*897*
**Medline**			
	#1	exp Hyperspectral Imaging/	221
	#2	exp Spectroscopy, Near-Infrared/	15,037
	#3	exp Spectrum Analysis, Raman/	24,798
	#4	exp Tomography, Optical Coherence/	41,925

	#5	(hyperspectral imaging or multispectral imaging or raman spectroscopy or diffuse reflectance spectroscopy or optical coherence tomography).ab,kf,ti.	77,334
	#6	exp Neoplasms/	3,625,535
	#7	(neoplasm or neoplasms or tumor or tumors or tumour or tumours or metastasis or metastases or glioma or gliomas).ab,kf,ti.	2,166,761
	#8	exp Brain/	1,280,937
	#9	exp Neurosurgery/	16,051
	#10	(brain or neurosurgery or intracranial).ab,kf,ti.	1,204,505
	#11	1 or 2 or 3 or 4 or 5	117,909
	#12	6 or 7	4,205,447
	#13	8 or 9 or 10	1,921,688
	#14	*11 and 12 and 13*	*485*

Table S3 Data extraction. A summary of the data that was extracted in the *Excel Microsoft Office 2020* data-extraction manual.

Category	*Data extracted*
**Article information**	title, year of publication, DOI, first author, journal, country.
**Optical method details**	optical method, system/provider, hand-held device or not, exposure time/time to data acquisition, working distance, number of spectra/images/pixels obtained, range of analyzed optical spectra.
**Tissue characteristics**	in vivo/ex vivo, preparation (if applicable), tumor/tissue type, number of patients, number of tumor/tissue samples, sample size.
**Study outcomes**	data processing/classification method, sensitivity, specificity, accuracy, other precision metrics, value of other precision metric, control.
**Comments**	study-specific comments.

Table S4. Characteristics of studies involving Raman Spectroscopy.

Study	Year	Country	Tumor (WHO grades)	In/ex vivo	Patients (tumor/normal)	Samples (tumor/normal)	Spectra (n)	Diagnostic algorithm
Riva et al. [20]	2021	Italy	OD II-III, A III, GBM IV	Ex vivo	-	63 (38/25)	3450 (2073/1377)	RF, Gb
Sciortino et al. [21]	2021	Italy	A II-III, OD II-III, GBM IV	Ex vivo	37	38	2073	RBF-SVM, XGB
Kopec et al. [22]	2021	Poland	MET, GS IV, AOD III, MEN II, MT I, PT, NF	Ex vivo	8	8	135600	PLS-DA
Jelke et al. [23]	2021	Luxembourg	MEN	Ex vivo	59 (48/11)	223	529 (422/107)	SVM
Pekmezci et al. [24]	2021	USA	GBM IV, AA III, AODIII, OD	Ex vivo	31	179	-	-
Aguiar et al. [25]	2020	Brazil	GBM, S, MB, MEN	Ex vivo	-	10	263	LDA, PLS-DA
Livermore et al. [26]	2020	UK	GBM, A II-III, AOD III, OD II, tumor resection cavity	Ex vivo	-	Part 1: 62; Part 2: 23	11624 (9799/1825)	PC-LDA
Bury et al. 1[27]	2020	UK	G I-IV, MEN I-III	Ex vivo	-	96 (88/8)	1911 (30 removed)	PCA-QDA
Hollon et al. 1 [28]	2020	USA	Unspecified	Ex vivo	278	-	-	CNN
Bovenkamp et al. [29]	2019	Austria	PT	Ex vivo	20	28 (28/0)	64,087	PCA, kNN
Sun et al. [30]	2019	China	G II-IV	Ex vivo	-	47 (23/24)	-	PLS, SVM, ANN
Morais et al. [31]	2019	UK	MEN I-II	Ex vivo	-	90 (90/0)	-	PCA-LDA,

								SPA-QDA
Galli et al. [32]	2019	Germany	G, MET, MEN, S, ”others”	Ex vivo	209	209 (202/7)	1070 (1033/37)	PCA
Uckermann et al. [33]	2018	Germany	G	Ex vivo	-	36	-	-
Bury et al. 2 [34]	2018	UK	MET	Ex vivo	-	21	-	PCA-LDC
Hollon et al. 2[35]	2018	USA	E, PA, circumscribed G, EP, GM, HB, DMG	Ex vivo	33	-	-	-
Jermyn et al. 1 integrated[36]	2017	Canada	MET, G II-IV	In vivo	15	161 (92/69) sites		Bt, SVM
Stables et al. [37]	2017	UK	GBM, MET	Ex vivo	41	48	952 (795/157)	KNN, SVM, LDA
Jermyn et al. 2 [38]	2016	Canada	A II-III, OD II-III, OA III, GBM IV	In vivo	13	105 (60/45)	-	-
Liu et al. [39]	2016	China	G	Ex vivo	20	-	133 (67/66)	-
Jermyn et al. 3 [40]	2015	Canada	A, OD, A, OD, OA, GBM, MET	In vivo	17	-	161 (95/66) (analysis)	Bt
Desroches et al. [41]	2015	Canada	G	In vivo	10	-	70 (58/12)	Bt
Ji et al. [42]	2015	USA	G	Ex vivo	22	-	-	GAM

Kalkanis et al. [43]	2014	USA	GBM	Ex vivo	17	-	3152	DFA
Bergner et al. [44]	2012	Germany	MEN	Ex vivo	-	22 (21/1)	-	Linear SVM, Radial SVM, PLS-DA
Auner et al. [45]	2012	USA	A, MB, ODG, E, AEP, GG	Ex vivo	-	19	435	DFA
Leslie et al. [46]	2012	USA	MB, E, OD, A, GG, “Other glioma”	Ex vivo	-	64 (31/33)	649 (321/328)	-
Kojenovic et al. 1 [47]	2005	Netherlands	MEN	Ex vivo	20	20	38 “mapping experiments”	PCA, KCA, LDA
Kojenovic et al. 2 [48]	2002	Netherlands	GBM	Ex vivo	20	20	24 “mapping experiments”	PCA, KCA, LDA

Abbreviations:
Tumors: A = Astrocytoma; AA = Anaplastic astrocytoma; AEP = Anaplastic ependymoma; AG = Astroganglioma; AOD = Anaplastic oligodendroglioma; E = Embryonal; EP = Ependymoma; G = Glioma; GBM = glioblastoma; GG = Ganglioglioma; GM = Germinoma; GS = Gliosarcoma; HB = Hemangioblastoma; M = Meningioma; MB = Medulloblastoma; Met = Metastasis; MT = Meningothelioma; N = Normal; NF = Neurofibroma; OA = Oligoastrocytoma; OD = Oligodendroglioma; PA = Pilocytic astrocytoma; and PT = Pituitary.
Diagnostic algorithms: ANN = Artificial Neural Network; Bt = Boosted trees; CNN = Convolutional Neural Network; DA = Discriminant analysis; DFA = Discriminant Function Analysis**;** DNN = Deep Neural Networks**;** GAM = Generalized additive model**;** Gb = Gradient Boosting**;** KCA = K-means Cluster Analysis**;** KNN = K- Nearest Neighbour classifier**;** kNN = kernel Neural Network**;** LDA = Linear Discriminant analysis**;** PC = Principal Components**;** PCA = Principal Component Analysis**;** PLS = Partial least squares**;** QDA = Quadrantic Discriminant Analysis**;** RBF = Radial Basis Function kernel**;** RF = Random Forrest**;** SPA = Successive Projections Algorithm; SVM = Support Vector Machine; and XGB = eXtreme Gradient Boosted trees.

Table S5. Study characteristics. Table listing the characteristics of all included studies investigating HSI.

Reference	Year	Country	Tumor (WHO grade)	In/Ex Vivo	Patient (n)	Sample (n)	Diagnostic algorithm
Urbanos et al. [49]	2021	Spain	G III, GBM	In vivo	12	-	SVM, RF, CNN
Manni et al. [50]	2020	Netherlands, Sweden	GBM IV	In vivo	16	-	2D-3D-CNN (hybrid)
Fabelo et al. 1 [51]	2019	Spain, USA	GBM	In vivo	16	-	2D-CNN, 1D-DNN
Fabelo et al. 2 [52]	2019	Spain, USA	GBM	In vivo	16	-	2D-CNN, 1D-DNN
Ortega et al. [53]	2018	Spain	GBM IV	Ex vivo	10	21	SVMs, ANNs, RFs

Abbreviations: Tumor: G = Glioma, GB = Glioblastoma multiforme
Diagnostic algorithms: ANN = Artificial Neural Networks, CNN = Convolutional Neural networks, DNN = Deep Neural Networks, RF = Random Forrest, SVM = Support Vector Machines

Table S6. Study characteristics. Table listing the characteristics of all included studies investigating OCT.

Reference	Year	Country	Tumor (WHO grade)	In/ex vivo	Patient (n)	Sample (n)	Images (n)	Diagnostic algorithm
Möller et al. [54]	2021	Germany	METs	Ex vivo	20	22	-	-
Yashin et al. [55]	2019	Russia	A II-III, GBM	In vivo/ex vivo	*Ex vivo:* 30	*Ex Vivo*: 176	*Ex Vivo:* 274	Visual assessment
Juarez-Chamb et al. [56]	2019	USA	G II-IV	In vivo	21	-	-	-
Kut et al. [57]	2015	USA	-	Ex vivo	37	128	4675	-

Abbreviations: A = Astrocytoma, G = Glioma, and MET = Metastases.

Table S7 Study characteristics. Table listing the characteristics of all included studies investigating DRS.

Reference	Year	Country	System	Tumor (WHO grade)	In/ex vivo	Patients (n)	Samples (n)	Measurements (n)	Diagnostic algorithm	Histopathology (HP); Normal Brain Tissue (NBT) (Yes/No)
Du Le et al. [58]	2017	Canada	Handheld fiber optic probe	GBM, low grade G	Ex vivo	7	22	-	-	HP: Yes, NBT: No
Lin et al. 1 [59]	2010	USA	Handheld fiber optic probe	PA, GG, CS, MB, high grade G, MAG	In vivo	12	-	59	-	HP: Yes, NBT: Yes
Majumder et al. [60]	2007	USA	custom designed fiber-optic probe (Visionex Inc.,Atlanta, GA)	-	In vivo	35	-	250	MRDF-SMLR, NMC	HP: Yes, NBT: Yes
Lin et al. 2 [61]	2001	USA	Handheld fiber optic probe	A, AG, GBM, OD METs	In vivo	26	-	120	-	HP: Yes, NBT: Yes

Abbreviations:
Tumors: A = Astrocytoma, AG = Astroganglioma, CS = Chondrosarcoma, G = Glioma, GBM = Glioblastoma multiforme GG = Ganglioglioma, MAG = Monomorphous angiocentric glioma, MB = Medulloblastoma, and MET = Metastases.
Diagnostic Algorithm: MRDF-SMLR = Maximum representation and discrimination feature-sparse multinominal logistic regression, and NMC = Nearest-mean classifier.

References:

1. C. V. Raman and K. S. Krishnan, "A new type of secondary radiation [11]," Nature **121**(3048), 501–502 (1928).
2. K. Kong, C. Kendall, N. Stone, and I. Notingher, "Raman spectroscopy for medical diagnostics — From in vitro biofluid assays to in vivo cancer detection," Adv Drug Deliv Rev **89**, 121–134 (2015).
3. R. R. Jones, D. C. Hooper, L. Zhang, D. Wolverson, and V. K. Valev, "Raman Techniques: Fundamentals and Frontiers," Nanoscale Res Lett **14**(1), (2019).
4. H. J. Butler, L. Ashton, B. Bird, G. Cinque, K. Curtis, J. Dorney, K. Esmonde-White, N. J. Fullwood, B. Gardner, P. L. Martin-Hirsch, M. J. Walsh, M. R. McAinsh, N. Stone, and F. L. Martin, "Using Raman spectroscopy to characterize biological materials," Nat Protoc **11**(4), 664–687 (2016).
5. D. DePaoli, É. Lemoine, K. Ember, M. Parent, M. Prud'homme, L. Cantin, K. Petrecca, F. Leblond, and D. C. Côté, "Rise of Raman spectroscopy in neurosurgery: a review," J Biomed Opt **25**(05), 1 (2020).
6. R. Kast, G. Auner, S. Yurgelevic, B. Broadbent, A. Raghunathan, L. M. Poisson, T. Mikkelsen, M. L. Rosenblum, and S. N. Kalkanis, "Identification of regions of normal grey matter and white matter from pathologic glioblastoma and necrosis in frozen sections using Raman imaging.," J Neurooncol **125**(2), 287–295 (2015).
7. J. Desroches, M. Jermyn, K. Mok, C. C. Lemieux-Leduc, J. Mercier, K. St-Arnaud, K. Urmey, M.-C. Guiot, E. Marple, K. Petrecca, and F. F. Leblond, "Characterization of a Raman spectroscopy probe system for intraoperative brain tissue classification.," Biomed Opt Express **6**(7), 2380–2397 (2015).
8. O. Uckermann, W. Yao, T. A. Juratli, R. Galli, E. Leipnitz, M. Meinhardt, E. Koch, G. Schackert, G. Steiner, and M. Kirsch, "IDH1 mutation in human glioma induces chemical alterations that are amenable to optical Raman spectroscopy," J Neurooncol **139**(2), 261–268 (2018).
9. D. A. Orringer, B. Pandian, Y. S. Niknafs, T. C. Hollon, J. Boyle, S. Lewis, M. Garrard, S. L. Hervey-Jumper, H. J. L. Garton, C. O. Maher, J. A. Heth, O. Sagher, D. A. Wilkinson, M. Snuderl, S. Venneti, S. H. Ramkissoon, K. A. McFadden, A. Fisher-Hubbard, A. P. Lieberman, T. D. Johnson, X. S. Xie, J. K. Trautman, C. W. Freudiger, and S. Camelo-Piragua, "Rapid intraoperative histology of unprocessed surgical specimens via fibre-laser-based stimulated Raman scattering microscopy," Nat Biomed Eng **1**(2), (2017).
10. T. Hollon and D. A. Orringer, "Label-free brain tumor imaging using Raman-based methods," J Neurooncol **151**(3), 393–402 (2021).
11. J. Shapey, Y. Xie, E. Nabavi, R. Bradford, S. R. Saeed, S. Ourselin, and T. Vercauteren, "Intraoperative multispectral and hyperspectral label-free imaging: A systematic review of in vivo clinical studies.," J Biophotonics **12**(9), e201800455 (2019).
12. L. Vignali, E. Solinas, and E. Emanuele, "Research and Clinical Applications of Optical Coherence Tomography in Invasive Cardiology: A Review," Curr Cardiol Rev **10**(4), 369–376 (2014).
13. B. Wan, C. Ganier, X. Du-Harpur, N. Harun, F. M. Watt, R. Patalay, and M. D. Lynch, "Applications and future directions for optical coherence tomography in dermatology*," British Journal of Dermatology **184**(6), 1014–1022 (2021).

14. Y. Fan, Y. Xia, X. Zhang, Y. Sun, J. Tang, L. Zhang, and H. Liao, "Optical coherence tomography for precision brain imaging, neurosurgical guidance and minimally invasive theranostics," Biosci Trends **12**(1), 12–23 (2018).
15. A. F. Fercher, C. K. Hitzenberger, W. Drexler, G. Kamp, and H. Sattmann, "In vivo optical coherence tomography," Am J Ophthalmol **116**(1), 113–114 (1993).
16. M. Ibne Mokbul, "Optical Coherence Tomography: Basic Concepts and Applications in Neuroscience Research," J Med Eng **2017**, 1–20 (2017).
17. A. G. Podoleanu, "Optical coherence tomography," J Microsc **247**(3), 209–219 (2012).
18. T. M. Bydlon, R. Nachabé, N. Ramanujam, H. J. C. M. Sterenborg, and B. H. W. Hendriks, "Chromophore based analyses of steady-state diffuse reflectance spectroscopy: current status and perspectives for clinical adoption," J Biophotonics **8**(1–2), 9–24 (2015).
19. S. Akter, M. G. Hossain, I. Nishidate, H. Hazama, and K. Awazu, "Medical applications of reflectance spectroscopy in the diffusive and sub-diffusive regimes:," https://doi.org/10.1177/0967033518806637 **26**(6), 337–350 (2018).
20. M. Riva, T. Sciortino, R. Secoli, E. D'Amico, S. Moccia, B. Fernandes, M. Conti Nibali, L. Gay, M. Rossi, E. De Momi, and L. Bello, "Glioma biopsies Classification Using Raman Spectroscopy and Machine Learning Models on Fresh Tissue Samples.," Cancers (Basel) **13**(5), (2021).
21. T. Sciortino, R. Secoli, E. d'Amico, S. Moccia, M. Conti Nibali, L. Gay, M. Rossi, N. Pecco, A. Castellano, E. De Momi, B. Fernandes, M. Riva, and L. Bello, "Raman Spectroscopy and Machine Learning for IDH Genotyping of Unprocessed Glioma Biopsies.," Cancers (Basel) **13**(16), (2021).
22. M. Kopec, M. Blaszczyk, M. Radek, and H. Abramczyk, "Raman imaging and statistical methods for analysis various type of human brain tumors and breast cancers.," Spectrochim Acta A Mol Biomol Spectrosc **262**, 120091 (2021).
23. F. Jelke, G. Mirizzi, F. K. Borgmann, A. Husch, R. Slimani, G. G. Klamminger, K. Klein, L. Mombaerts, J.-J. Gérardy, M. Mittelbronn, and F. Hertel, "Intraoperative discrimination of native meningioma and dura mater by Raman spectroscopy.," Sci Rep **11**(1), 23583 (2021).
24. M. Pekmezci, R. A. Morshed, P. Chunduru, B. Pandian, J. Young, J. E. Villanueva-Meyer, T. Tihan, E. A. Sloan, M. K. Aghi, A. M. Molinaro, M. S. Berger, and S. L. Hervey-Jumper, "Detection of glioma infiltration at the tumor margin using quantitative stimulated Raman scattering histology.," Sci Rep **11**(1), 12162 (2021).
25. R. P. Aguiar, E. T. Falcão, C. A. Pasqualucci, L. J. Silveira, E. T. Falcao, C. A. Pasqualucci, and L. J. Silveira, "Use of Raman spectroscopy to evaluate the biochemical composition of normal and tumoral human brain tissues for diagnosis," Lasers Med Sci **37**(1), 121–133 (2020).
26. L. J. Livermore, M. Isabelle, I. M. Bell, O. Edgar, N. L. Voets, R. Stacey, O. Ansorge, C. Vallance, and P. Plaha, "Raman spectroscopy to differentiate between fresh tissue samples of glioma and normal brain: a comparison with 5-ALA-induced fluorescence-guided surgery," J Neurosurg **132**(2), 469–479 (2021).
27. D. Bury, C. L. M. Morais, F. L. Martin, K. M. G. Lima, K. M. Ashton, M. J. Baker, and T. P. Dawson, "Discrimination of fresh frozen non-tumour and tumour brain tissue using spectrochemical analyses and a classification model.," Br J Neurosurg **34**(1), 40–45 (2020).
28. T. C. Hollon, B. Pandian, A. R. Adapa, E. Urias, A. V Save, S. S. S. Khalsa, D. G. Eichberg, R. S. D'Amico, Z. U. Farooq, S. Lewis, P. D. Petridis, T. Marie, A. H. Shah, H. J. L. Garton, C. O. Maher, J. A. Heth, E. L. McKean, S. E. Sullivan, S. L. Hervey-Jumper, P. G. Patil, B. G. Thompson, O. Sagher, G. M. 2nd McKhann, R. J. Komotar,

M. E. Ivan, M. Snuderl, M. L. Otten, T. D. Johnson, M. B. Sisti, J. N. Bruce, K. M. Muraszko, J. Trautman, C. W. Freudiger, P. Canoll, H. Lee, S. Camelo-Piragua, and D. A. Orringer, "Near real-time intraoperative brain tumor diagnosis using stimulated Raman histology and deep neural networks.," Nat Med **26**(1), 52–58 (2020).
29. D. Bovenkamp, A. Micko, J. Puls, F. Placzek, R. Hoftberger, G. Vila, R. Leitgeb, W. Drexler, M. Andreana, S. Wolfsberger, and A. Unterhuber, "Line Scan Raman Microspectroscopy for Label-Free Diagnosis of Human Pituitary Biopsies.," Molecules **24**(19), (2019).
30. J. J. Sun, H. Fang, Z. Q. Zhang, M. L. Chen, J. J. Tian, L. Chen, X. Zou, H. C. Yin, and J. Yin, "Detection of glioma by surface-enhanced Raman scattering spectra with optimized mathematical methods," JOURNAL OF RAMAN SPECTROSCOPY **50**(8), 1130–1140 (2019).
31. C. L. M. Morais, T. Lilo, K. M. Ashton, C. Davis, T. P. Dawson, N. Gurusinghe, and F. L. Martin, "Determination of meningioma brain tumour grades using Raman microspectroscopy imaging.," Analyst **144**(23), 7024–7031 (2019).
32. R. Galli, M. Meinhardt, E. Koch, G. Schackert, G. Steiner, M. Kirsch, and O. Uckermann, "Rapid Label-Free Analysis of Brain Tumor Biopsies by Near Infrared Raman and Fluorescence Spectroscopy-A Study of 209 Patients.," Front Oncol **9**, 1165 (2019).
33. O. Uckermann, W. Yao, T. A. Juratli, R. Galli, E. Leipnitz, M. Meinhardt, E. Koch, G. Schackert, G. Steiner, and M. Kirsch, "IDH1 mutation in human glioma induces chemical alterations that are amenable to optical Raman spectroscopy," J Neurooncol **139**(2), 261–268 (2018).
34. D. Bury, G. Faust, M. Paraskevaidi, K. M. Ashton, T. P. Dawson, and F. L. Martin, "Phenotyping Metastatic Brain Tumors Applying Spectrochemical Analyses: Segregation of Different Cancer Types," Anal Lett **52**(4), 575–587 (2019).
35. T. C. Hollon, S. Lewis, B. Pandian, Y. S. Niknafs, M. R. Garrard, H. Garton, C. O. Maher, K. McFadden, M. Snuderl, A. P. Lieberman, K. Muraszko, S. Camelo-Piragua, and D. A. Orringer, "Rapid Intraoperative Diagnosis of Pediatric Brain Tumors Using Stimulated Raman Histology.," Cancer Res **78**(1), 278–289 (2018).
36. J. Desroches, M. Jermyn, M. Pinto, F. Picot, M.-A. Tremblay, S. Obaid, E. Marple, K. Urmey, D. Trudel, G. Soulez, M.-C. Guiot, B. C. Wilson, K. Petrecca, and F. Leblond, "A new method using Raman spectroscopy for in vivo targeted brain cancer tissue biopsy.," Sci Rep **8**(1), 1792 (2018).
37. R. Stables, G. Clemens, H. J. Butler, K. M. Ashton, A. Brodbelt, T. P. Dawson, L. M. Fullwood, M. D. Jenkinson, and M. J. Baker, "Feature driven classification of Raman spectra for real-time spectral brain tumour diagnosis using sound," ANALYST **142**(1), 98–109 (2017).
38. M. Jermyn, J. Desroches, J. Mercier, K. St-Arnaud, M. C. Guiot, F. Leblond, and K. Petrecca, "Raman spectroscopy detects distant invasive brain cancer cells centimeters beyond MRI capability in humans," Biomed Opt Express **7**(12), 5129–5137 (2016).
39. T. Liu, C. S. Chen, X. Z. Shi, and C. Y. Liu, "Evaluation of Raman spectra of human brain tumor tissue using the learning vector quantization neural network," Laser Phys **26**(5), (2016).
40. M. Jermyn, K. Mok, J. Mercier, J. Desroches, J. Pichette, K. Saint-Arnaud, L. Bernstein, M.-C. Guiot, K. Petrecca, and F. Leblond, "Intraoperative brain cancer detection with Raman spectroscopy in humans.," Sci Transl Med **7**(274), 274ra19 (2015).
41. J. Desroches, M. Jermyn, K. Mok, C. C. Lemieux-Leduc, J. Mercier, K. St-Arnaud, K. Urmey, M.-C. Guiot, E. Marple, K. Petrecca, and F. F. F. Leblond, "Characterization

of a Raman spectroscopy probe system for intraoperative brain tissue classification," Biomed Opt Express **6**(7), 2380 (2015).

42. M. Ji, S. Lewis, S. Camelo-Piragua, S. H. Ramkissoon, M. Snuderl, S. Venneti, A. Fisher-Hubbard, M. Garrard, D. Fu, A. C. Wang, J. A. Heth, C. O. Maher, N. Sanai, T. D. Johnson, C. W. Freudiger, O. Sagher, X. S. Xie, and D. A. Orringer, "Detection of human brain tumor infiltration with quantitative stimulated Raman scattering microscopy.," Sci Transl Med **7**(309), 309ra163 (2015).
43. S. N. Kalkanis, R. E. Kast, M. L. Rosenblum, T. Mikkelsen, S. M. Yurgelevic, K. M. Nelson, A. Raghunathan, L. M. Poisson, and G. W. Auner, "Raman spectroscopy to distinguish grey matter, necrosis, and glioblastoma multiforme in frozen tissue sections.," J Neurooncol **116**(3), 477–485 (2014).
44. N. Bergner, B. F. M. Romeike, R. Reichart, R. Kalff, C. Krafft, and J. U. Popp, "Tumor margin identification and prediction of the primary tumor from brain metastases using FTIR imaging and support vector machines," ANALYST **138**(14), 3983–3990 (2013).
45. A. W. Auner, R. E. Kast, R. Rabah, J. M. Poulik, and M. D. Klein, "Conclusions and data analysis: a 6-year study of Raman spectroscopy of solid tumors at a major pediatric institute.," Pediatr Surg Int **29**(2), 129–140 (2013).
46. D. G. Leslie, R. E. Kast, J. M. Poulik, R. Rabah, S. Sood, G. W. Auner, M. D. Klein, R. E. Kast, R. Rabah, L. Reisner, A. Pandya, S. Sood, G. W. Auner, and M. D. Klein, "IDENTIFICATION OF PEDIATRIC BRAIN NEOPLASMS USING RAMAN SPECTROSCOPY," Neuro Oncol **12**(14th International Symposium on Pediatric Neuro-Oncology), II52–II52 (2010).
47. S. Koljenović, T. B. Schut, A. Vincent, J. M. Kros, G. J. Puppels, S. Koljenovic, T. B. Schut, A. Vincent, J. M. Kros, and G. J. Puppels, "Detection of meningioma in dura mater by Raman spectroscopy," Anal Chem **77**(24), 7958–7965 (2005).
48. S. Koljenović, L.-P. Choo-Smith, T. C. Bakker Schut, J. M. Kros, H. J. van den Berge, G. J. Puppels, S. Koljenovic, L.-P. Choo-Smith, T. C. Bakker Schut, J. M. Kros, H. J. van den Berge, G. J. Puppels, S. Koljenović, L.-P. Choo-Smith, T. C. Bakker Schut, J. M. Kros, H. J. van den Berge, and G. J. Puppels, "Discriminating vital tumor from necrotic tissue in human glioblastoma tissue samples by Raman spectroscopy.," Lab Invest **82**(10), 1265–1277 (2002).
49. G. Urbanos, A. Martin, G. Vazquez, M. Villanueva, M. Villa, L. Jimenez-Roldan, M. Chavarrias, A. Lagares, E. Juarez, and C. Sanz, "Supervised Machine Learning Methods and Hyperspectral Imaging Techniques Jointly Applied for Brain Cancer Classification.," Sensors (Basel) **21**(11), (2021).
50. F. Manni, F. van der Sommen, H. Fabelo, S. Zinger, C. Shan, E. Edstrom, A. Elmi-Terander, S. Ortega, G. Marrero Callico, and P. H. N. de With, "Hyperspectral Imaging for Glioblastoma Surgery: Improving Tumor Identification Using a Deep Spectral-Spatial Approach.," Sensors (Basel) **20**(23), (2020).
51. H. Fabelo, M. Halicek, S. Ortega, M. Shahedi, A. Szolna, J. F. Pineiro, C. Sosa, A. J. O'Shanahan, S. Bisshopp, C. Espino, M. Marquez, M. Hernandez, D. Carrera, J. Morera, G. M. Callico, R. Sarmiento, and B. Fei, "Deep Learning-Based Framework for In Vivo Identification of Glioblastoma Tumor using Hyperspectral Images of Human Brain.," Sensors (Basel) **19**(4), (2019).
52. H. Fabelo, M. Halicek, S. Ortega, A. Szolna, J. Morera, R. Sarmiento, G. M. Callico, and B. Fei, "Surgical Aid Visualization System for Glioblastoma Tumor Identification based on Deep Learning and In vivo Hyperspectral Images of Human Patients.," Proc SPIE Int Soc Opt Eng **10951**, (2019).

53. S. Ortega, H. Fabelo, R. Camacho, M. de la Luz Plaza, G. M. Callico, and R. Sarmiento, "Detecting brain tumor in pathological slides using hyperspectral imaging.," Biomed Opt Express **9**(2), 818–831 (2018).
54. J. Möller, A. Bartsch, M. Lenz, I. Tischoff, R. Krug, H. Welp, M. R. Hofmann, K. Schmieder, D. Miller, J. Moller, A. Bartsch, M. Lenz, I. Tischoff, R. Krug, H. Welp, M. R. Hofmann, K. Schmieder, D. Miller, J. Möller, A. Bartsch, M. Lenz, I. Tischoff, R. Krug, H. Welp, M. R. Hofmann, K. Schmieder, and D. Miller, "Applying machine learning to optical coherence tomography images for automated tissue classification in brain metastases.," Int J Comput Assist Radiol Surg **16**(9), 1517–1526 (2021).
55. K. S. Yashin, E. B. Kiseleva, E. V Gubarkova, A. A. Moiseev, S. S. Kuznetsov, P. A. Shilyagin, G. V Gelikonov, I. A. Medyanik, L. Y. Kravets, A. A. Potapov, E. V Zagaynova, and N. D. Gladkova, "Cross-Polarization Optical Coherence Tomography for Brain Tumor Imaging.," Front Oncol **9**, 201 (2019).
56. R. M. Juarez-Chambi, C. Kut, J. J. Rico-Jimenez, K. L. Chaichana, J. Xi, D. U. Campos-Delgado, F. J. Rodriguez, A. Quinones-Hinojosa, X. Li, and J. A. Jo, "AI-Assisted In Situ Detection of Human Glioma Infiltration Using a Novel Computational Method for Optical Coherence Tomography.," Clin Cancer Res **25**(21), 6329–6338 (2019).
57. C. Kut, K. L. Chaichana, J. Xi, S. M. Raza, X. Ye, E. R. McVeigh, F. J. Rodriguez, A. Quinones-Hinojosa, and X. Li, "Detection of human brain cancer infiltration ex vivo and in vivo using quantitative optical coherence tomography.," Sci Transl Med **7**(292), 292ra100 (2015).
58. V. N. Du Le, J. Provias, N. Murty, M. S. Patterson, Z. J. Nie, J. E. Hayward, T. J. Farrell, W. McMillan, W. B. Zhang, and Q. Y. Fang, "Dual-modality optical biopsy of glioblastomas multiforme with diffuse reflectance and fluorescence: ex vivo retrieval of optical properties," J Biomed Opt **22**(2), (2017).
59. W.-C. Lin, D. I. Sandberg, S. Bhatia, M. Johnson, S. Oh, and J. Ragheb, "Diffuse reflectance spectroscopy for in vivo pediatric brain tumor detection.," J Biomed Opt **15**(6), 61709 (2010).
60. S. K. Majumder, S. Gebhart, M. D. Johnson, R. Thompson, W. C. Lin, and A. Mahadevan-Jansen, "A probability-based spectroscopic diagnostic algorithm for simultaneous discrimination of brain tumor and tumor margins from normal brain tissue," Appl Spectrosc **61**(5), 548–557 (2007).
61. W.-C. Lin, S. A. Toms, M. Johnson, E. D. Jansen, and A. Mahadevan-Jansen, "In Vivo Brain Tumor Demarcation Using Optical Spectroscopy¶," Photochem Photobiol **73**(4), 396 (2001).
